# A Brief Review on New Naturally Occurring Cembranoid Diterpene Derivatives from the Soft Corals of the Genera *Sarcophyton*, *Sinularia*, and *Lobophytum* Since 2016

**DOI:** 10.3390/molecules24040781

**Published:** 2019-02-21

**Authors:** Inna Glibka Rodrigues, Maria Graça Miguel, Wissem Mnif

**Affiliations:** 1 Faculdade de Ciências e Tecnologia, Departamento de Química e Farmácia, Universidade do Algarve, Edifício 8, MeditBio, Campus de Gambelas, 8005-139 Faro, Portugal; innaglibkarodrigues@gmail.com (I.G.R.); mgmiguel@ualg.pt (M.G.M.); 2 Department of Chemistry, Faculty of Sciences and Arts in Balgarn, University of Bisha, Bisha 61922, P.O. BOX 199, Saudi Arabia; 3 Univ. Manouba, ISBST, BVBGR-LR11ES31, Biotechpole Sidi Thabet, Ariana 2020, Tunisia

**Keywords:** *Sarcophyton*, *Sinularia*, *Lobophytum*, anti-microbial, anti-inflammatory, anti-tumoral

## Abstract

This work reviews the new isolated cembranoid derivatives from species of the genera *Sarcophyton*, *Sinularia*, and *Lobophytum* as well as their biological properties, during 2016–2018. The compilation permitted to conclude that much more new cembranoid diterpenes were found in the soft corals of the genus *Sarcophyton* than in those belonging to the genera *Lobophytum* or *Sinularia*. Beyond the chemical composition, the biological properties were also reviewed, namely anti-microbial against several Gram-positive and Gram-negative bacteria and fungi, anti-inflammatory and anti-tumoral against several types of cancer cells. In spite of the biological activities detected in almost all samples, there is a remarkable diversity in the results which may be attributed to the chemical variability that needs to be deepened in order to develop new molecules with potential application in medicine.

## 1. Introduction

**Figure 1:**
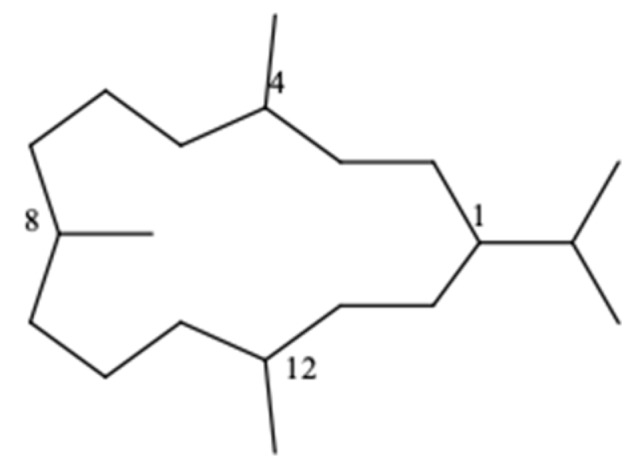
Cembrane skeleton.

The cembrane skeleton is isoprenoid and consists of a fourteen-membered carbocyclic ring with an isopropyl residue at position 1 and three methyl groups at positions 4, 8, and 12 (Figure 1). The basic structure of this diterpene usually presents cyclic ether, lactone, or furan moieties around the macrocyclic ring. There are also cembranoids variants which contain a 12 or 13-membered carbon skeleton [1,2,3].

In nature, this class of diterpenoids has been found in marine invertebrates, lower and higher plants, insects (termites), and even paracloacal glands of Chinese male alligators (*Alligator sinensis*) [4,5]. Cembranoids from marine invertebrates are particularly isolated from soft corals of the genera *Sinularia, Lobophytum*, *Eunicea*, *Clavularia*, and *Sarcophyton*, and from the gorgonian octocorals, mainly of the genera *Pseudopterogorgia*, *Leptogorgia*, and *Lophogorgia* [1,6,7].

Soft corals (phylum, Cnidaria; class, Anthozoa; subclass, Octocorallia; order, Alcyonaceae; family, Alcyoniidae) have been the target of study since the nineteenth century. The subclass Octocorallia includes soft corals, gorgonians, and sea pens. Most soft corals belong to the order Alcyonacea that comprises several families, including Alcyoniidae. This family contains the genera *Sarcophyton*, *Sinularia*, and *Lobophytum* [8]. Soft corals are found in Indo Pacific reefs whereas Gorgonian octocorals dominate the biomass in coral reef environments of the north-western Atlantic Ocean and in the Caribbean Sea [7].

In nature, cembranoids may act as chemical defense compounds against fish predators and/or competing for reef organisms, bacteria, parasites, to ensure their protection and survival [7,9]. Multiple in vitro biological properties of cembranoids of marine origin have been reported such as anti-inflammatory, anti-tumoral, anti-bacterial, anti-viral, neuroprotective, antiarthritic, calcium-antagonistic, and cytotoxic [9,10]. This is the first step for the in vivo assays which will determine whether or not they constitute potential therapeutic agents.

Yang et al. [10] review all the metabolites of cembrane diterpenes either from terrestrial or marine organisms up to 2010. They were divided into several different families according to the variety of ring sizes, oxidation patterns, and the respective biological activities. Several other reviews have been made regarding new compounds and their biological activities. These compounds have been isolated from marine microorganisms and phytoplankton, green, brown and red algae, sponges, cnidarians, bryozoans, mollusks, tunicates, echinoderms, mangroves and other intertidal plants, from 2013 until 2017 [11,12,13,14]. Marine invertebrates isolated from soft corals of the genera *Sinularia*, *Lobophytum*, *Eunicea*, and *Sarcophyton* are also included in these reviews.

Liang and Guo [15], in a review on the terpenes from the soft coral of the genus *Sarcophyton* (*S. elegans*, *S. glaucum*, *S. ehrenbergi*, *S. trocheliophorum*, *S. molle*, *S. mililatensis*, *S. crassocaule*, *S. latum*, *S. cherbonnieri*, *S. stolidotum*, *S. tortuosum*, *S. infundibuliforme*, *S. flexuosum*, *S. solidum*, and some undefined species) from different geographical origins, reported 165 diterpenes, 29 biscembranoids, among other terpene compounds, during the period 1995–July 2011. Some of these compounds possessed biological properties. 

The present work will review the new cembranoid diterpenes isolated from species belonging to the family Alcyoniidae, which contains the genera *Sarcophyton*, *Sinularia*, and *Lobophytum* as well as their biological properties, since 2016. For this review, only the *Web of Science* was used as a database for research by utilizing the keywords *cembrane*, and *cembranoid*.

## 2. Chemical Structure of Cembranoids from Marine Origin

According to Rodríguez et al. [16], the cembrane skeleton of marine origin is derived from the cyclization of geranylgeranyl pyrophosphate. This hypothesis is based on the fact that the double bonds of the cembrane skeleton have the geometry *E* which is observed in geranylgeraniol.

Cembrane diterpenoids have diverse structural variations with a multitude of functional groups (lactone, epoxide, furan, ester, aldehyde, hydroxyl, carboxyl moieties) and cyclizations, which permit to group them in several families [10,17]. According to the review of Yang et al. [10], the cembrane-type diterpenoids may be classified as depicted in Table 1.

Cembranolides possess a 14-membered carbocyclic nucleus, generally fused to a 5-, 6-, 7-, or 8-membered lactone ring. Furanocembranoids possess a 14-membered carbocyclic nucleus as well as a furan heterocycle. They also have a butenolide moiety involving C-10–C-12, and C-20. Biscembranoids possess a 14-6-14 membered tricyclic backbone of tetraterpenoids [10]. The structure of polymaxenolide (**13**) comprises a 14-membered cembranoid skeleton linked via a spiro ring system, to an africanane skeleton (Table 1) [18].

There are also the polycyclic norcembranoid diterpenes, rare and found exclusively in soft corals of the genus *Sinularia*. These diterpenes are within the family of furanecembranoids which lack a C-18 carbon substituent in comparison with C20-cembranoids. They co-occur with 14-membered macrocyclic norcembranoids with a furan heterocycle in which also lacks a C-18 carbon substituent [19]. The mechanisms leading to the occurrence of norcembranoid diterpenes are not well understood but they may include the production of anionic and radical intermediates along with competitive transannular carbon-to-carbon bond-forming reactions. However, these are only proposals that, according to the authors [19], must be validated in forthcoming biosynthetic studies. 

The extraction of cembranoid diterpenes was generally made with organic solvents (acetone, chloroform, ethanol, ethyl acetate, methanol, and methylene chloride) by maceration (Table 2, Table 3 and Table 4), followed by the concentration under vacuum. Afterwards, the residue is partitioned between pairs of solvents and further column chromatography eluting with a gradient of solvents with increasing polarity. Different fractions originate the cembranoid compounds, which can be subjected to semi- or preparative HPLC (high performance liquid chromatography). The identification of compounds is generally made through ^1^H-NMR (proton nuclear magnetic resonance), ^13^C-NMR (Carbon-13 nuclear magnetic resonance), one dimensional and two dimensional nuclear magnetic resonance (1D-NMR and 2D-NMR) including ^1^H-^1^H COSY, HMQC, HMBC, and NOESY spectra (Correlation Spectroscopy, Heteronuclear Multiple-Quantum Correlation, Heteronuclear Multiple-Bond Correlation Spectroscopy, Nuclear Overhauser Effect Spectroscopy, respectively), Time-Dependent Density Functional Theory Electronic Circular Dichroism (TDDFT/ECD), Density Functional Theory (DFT)/NMR calculations, FTIR (Fourier-transform infrared spectrosocopy), single crystal X-ray diffraction, and LC-MS-IT-TOF (liquid chromatography–mass spectrometry-ion trap-time-of-flight) [9,20,21,22,23,24,25,26,27,28,29,30,31,32,33,34,35,36,37,38,39,40,41,42,43,44,45,46,47,48,49,50,51,52,53,54,55,56,57].

The review upon the source, chemistry and bioactivities of new cembrane diterpenes from marine organisms, since 2016 (27 works) (Table 2, Table 3 and Table 4) revealed that the most important sources of cembrane derivatives found in that period were coming from the genus *Sarcophyton* (14 works), *Sinalarina* (8 works) and *Lobophytum* (5 works). There is still one work in which the authors did not isolate new cembrane compounds but they checked the biological properties of the crude methanolic extract of *Lobophytum crassum* from the coast of Madagascar [24]. In other work, Al-Footy et al. [52] reported that among diverse secondary metabolites isolated from the soft coral *Lobophytum* sp. collected off the Red Sea Coast, in Jeddah, Saudi Arabia, only the known cembrane diterpenoid (cembrene A) had an antibacterial activity against several Gram-positive and Gram-negative microorganisms. For this reason, the brief review aims at identifying the new compounds found in those species belonging to these genera, during that period.

## 3. New Cembrane Derivatives from the Genus *Sarcophyton*

The genus *Sarcophyton* presents a large number of species. Many of these species have been chemically examined. Some examples of groups of compounds include sesquiterpenes, diterpenes, diterpene dimers, prostaglandins, steroids, and ceramides, which have been extensively reviewed [10,11,12,13,14,15,17,58]. Among those metabolites, terpenes are the most frequently detected, possessing many biological properties (anti-inflammatory, anti-viral, anti-fouling, cytotoxic, neuroprotective) according to the review made by Yang et al. [10].

Fourteen works regarding new cembranoid diterpenes from the genus *Sarcophyton* (*S. cherbonnieri*, *S. ehrenbergi*, *S. elegans*, *S. stellatum*, *S. subviride*, and *S. trocheliophorum*) were found during the last three years. These species of soft corals were collected at several places: seven samples in the South China Sea (one *Sarcophyton* sp., one *S. ehrenbergi*, one *S. elegans*, one *S. stellatum*, one *S. subviride* and two *S. trocheliophorum*); two samples of *Sarcophyton* sp. in the Celebes Sea; one sample of S. *stellatum* in the Indian Ocean; one sample of *S. cherbonnieri* in the Philippine Sea; and three samples collected in the Red Sea Coast (one S. *ehrenbergi* and two S. *trocheliophorum*) (Table 2).

There is a work in which the isolation and identification of metabolites were not performed. Only the antimicrobial and cytotoxicity activities of extracts of two soft corals (*Lobophytum microlobulatum*, *Sarcophyton auritum*), three seaweeds (*Caulerpa racemosa*, *Caulerpa sertularioides*, *Kappaphycus alvarezii*), and a marine sponge (*Spheciospongia vagabunda*) collected from Malaysian coast were determined. Hexane extract of *Sarcophyton auritum* exhibited strong fungicidal activity against dimorphic yeast *Cryptococcus neoformans*, with minimal inhibitory concentration (MIC) and minimum fungicidal concentration (MFC) values of 0.04 mg/mL (in both). The ethyl acetate extract of *S. auritum* showed strong inhibition on the cytopathic effect induced by the *Chikungunya* virus (a re-emerging mosquito-borne virus) with 50% effective concentrations of 176.6 +/− 9.7 mu g/mL. According to Chan et al. [59], extracts from the two soft corals (*L. microlobulatum* and *S. auritum*) possessed stronger antimicrobial activity than the seaweeds and the sponge.

Beyond the publications regarding the discovery of new cembrane diterpenoids in the genus *Sarcophyton* as well as their biological properties, three other publications with distinct approaches were found. One of them aimed at examining the effect of oxylipin analogues [prostaglandin E1 (PG-E1), methyl jasmonate, and arachidonic acid in addition to the geranylgeranylpyrophosphate] and wounding on the secondary metabolism of the soft corals *Sarcophyton glaucum* and *Lobophyton pauciflorum* [60]. According to the authors, the PG-E1 was more effective for upregulating campestene-triol and a cembranoid than methyl jasmonate in the soft corals *Sarcophyton glaucum*. In addition, the effect of the elicitors in *Lobophyton pauciflorum* was poorer than that in *Sarcophyton glaucum* [60]. The second one applied the quantitative NMR (qNMR) for assessing the diterpene variation in 16 soft coral specimens in the context of their genotype, origin, and growing habitat. The study revealed higher diterpene amounts in *Sarcophyton* sp. than in *Sinularia* or *Lobophyton* [29]. In their publication, Farag et al. [29] reported the metabolite profile of the soft coral genus *Sarcophyton* in different habitats along the coastal Egyptian Red Sea, was performed through ^1^H-NMR and ultra-performance liquid chromatography-mass spectrometry (UPLC-MS). At the same time, the authors compared the metabolite profile of these wild soft corals with those growing in aquarium. Generally, wild soft corals presented more bioactive compounds than aquarium grown ones. This discrepancy found between wild and aquarium grown corals were attributed, by the authors, to the lack of necessity for producing compounds acting as defenses against predators absent in tanks.

The large-scale metabolomics analyses were made for the first time in 16 *Sarcophyton* species, comparing MS (mass spectra) and NMR results. The metabolomic fingerprinting and profiling of those soft coral extracts were made through 1D and 2D-NMR without any preliminary chromatographic assay. In parallel to the chromatographic mass spectrometry techniques, permitted to identify 120 metabolites including 65 diterpenes, 8 sesquiterpenes, 18 sterols, and 15 oxylipids. The authors have used statistical multivariate analyses such as principal component analysis (PCA) and orthogonal projection to latent structures-discriminant analysis (OPLS-DA) for samples classification [30]. In the same work, they concluded that UPLC-MS (ultra-performance liquid chromatography-mass spectrometry) revealed to be better tool for a compound based classification of coral species than NMR technique. However, NMR or UPLC−MS data sets were likewise effective in foreseeing the species origin of unknown *Sarcophyton* after applying PCA [30]. 

**Figure 2:**
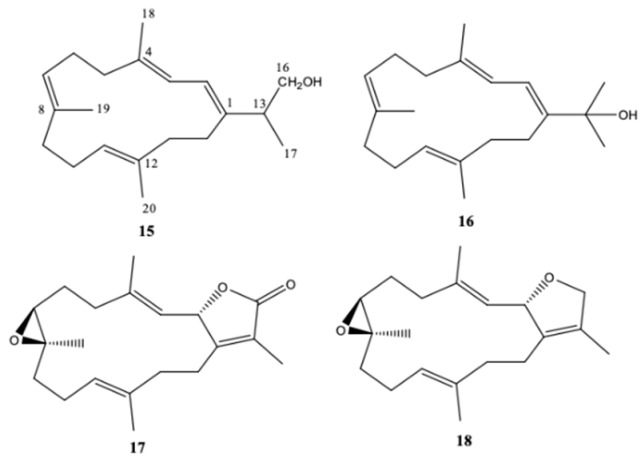
Cembranoid diterpenes isolated from *Sarcophyton* sp., collected at the Karah Island (West Malaysia) [21].

Kamada et al. [21] from a Malaysian specimen of *Sarcophyton* sp., collected at the Karah Island (West Malaysia), isolated, identified and evaluated the antibacterial activity of 16-hydroxy-cembra-1,3,7,11-tetraene (**15**) (Figure 2), a new cembrane, along with the known cembrane diterpenes 15-hydroxycembra-1,3,7,11-tetraene (**16**), sarcophine (**17**), and sarcophytoxide (**18**). The antimicrobial activity of all compounds was assayed against antibiotic resistant clinical bacterial strains *Staphylococcus aureus* and *Escherichia coli*. Only the new compound presented inhibition against *Staphylococcus aureus*. Its MBC (Minimum Bactericidal Concentration) and MIC (Minimum Inhibitory Concentration) were 75 and 25 μg/mL, respectively [21].

**Figure 3:**
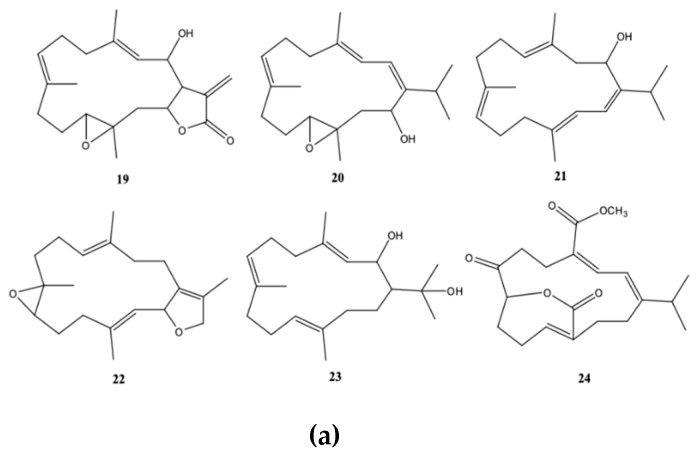

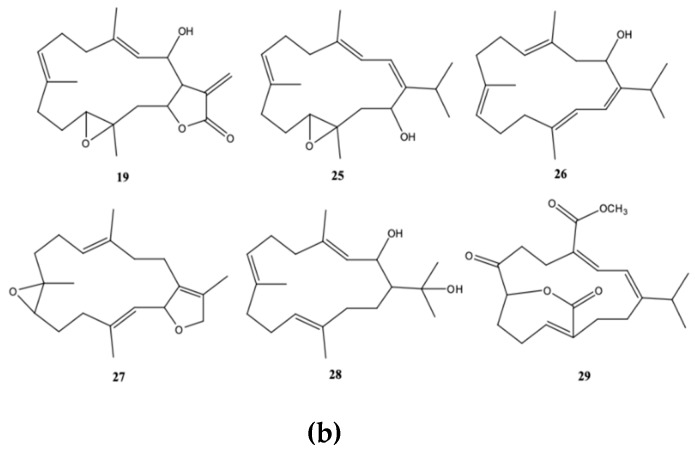
Cembranoid diterpenes isolated from *Sarcophyton* sp. on the reef at Mahengetang Island (Indonesia) [31]. (**a**) The names are attributed according to the authors: sarcophytoxide (**20**), sarcrassin E (**21**), 3,7,11-cembretriene-2,15-diol (**22**), 11,12-epoxy-sarcophytol A (**23**), and sarcophytol A (**24**); (**b**) names attributed to the literature (11,12-epoxysarcophytol A (**25**), sarcophytol A (**26**), sarcophytoxide (**27**), 3,7,11-cembretriene-2,15-diol (**28**), and sarcrassin E (**29**).

From dominant soft coral species of the genus *Sarcophyton* sp. on the reef at Mahengetang Island (Indonesia), Januar et al. [31] isolated a new compound 2-hydroxy-crassocolide E (**19**) alongside with 5 known cembranoid compounds sarcophytoxide (**20**), sarcrassin E (**21**), 3,7,11-cembretriene-2,15-diol (**22**), 11,12-epoxy-sarcophytol A (**23**), and sarcophytol A (**24**) (Figure 3a). However, and according to the structures presented by the authors, the known cembranoid compounds should be, by the same order, 11,12-epoxysarcophytol A (**25**), sarcophytol A (**26**), sarcophytoxide (**27**), 3,7,11-cembretriene-2,15-diol (**28**), and sarcrassin E (**29**) (Figure 3b). All compounds inhibited the growth of human breast tumor cell lines MCF-7, being the IG_50_ (inhibition growth 50) value of 18.3 ppm for the new compound [31].

**Figure 4:**
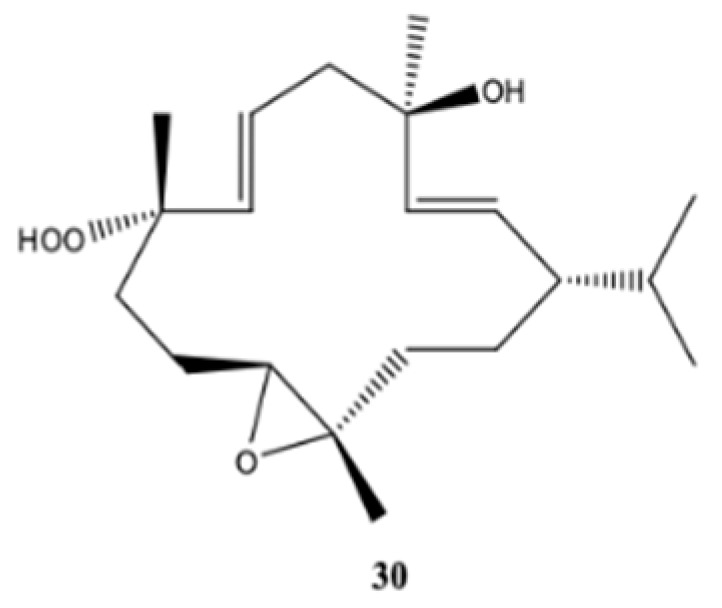
New cembranoid diterpene isolated from *Sarcophyton* sp. collected from the coastal waters Bohey Dulang, Sabah, Malaysia [32].

Kamada et al. [32] isolated and identified one new cembrane diterpene, 1*S*,2*E*,4*R*,6*E*,8*S*,11*S*,12*S*)-11,12-epoxy-8-hydroperoxy-4-hydroxy-2,6-cembradiene (**30**) (Figure 4), from a population of soft coral genus *Sarcophyton* sp. collected from the coastal waters Bohey Dulang, Sabah, Malaysia. This compound did not exhibit cytotoxic activity against human promyelocytic leukemia cells (HL-60) (IC_50_ > 30 μg/mL). The same compound was not able to prevent the accumulation of nitric oxide (NO), prostaglandin E_2_ (PGE_2_) and pro-inflammatory cytokines (TNF-α, IL-1β and IL-6) in lipopolysaccharide (LPS)-induced mouse leukaemic monocyte macrophage (RAW 264.7 cells), that is, it did not possess anti-inflammatory activity. In contrast, the new compound showed strong activity against the seaweed pathogens *Alteromonas* sp., *Cytophaga-Flavobacterium* and *Vibrio* sp. [32].

**Figure 5:**
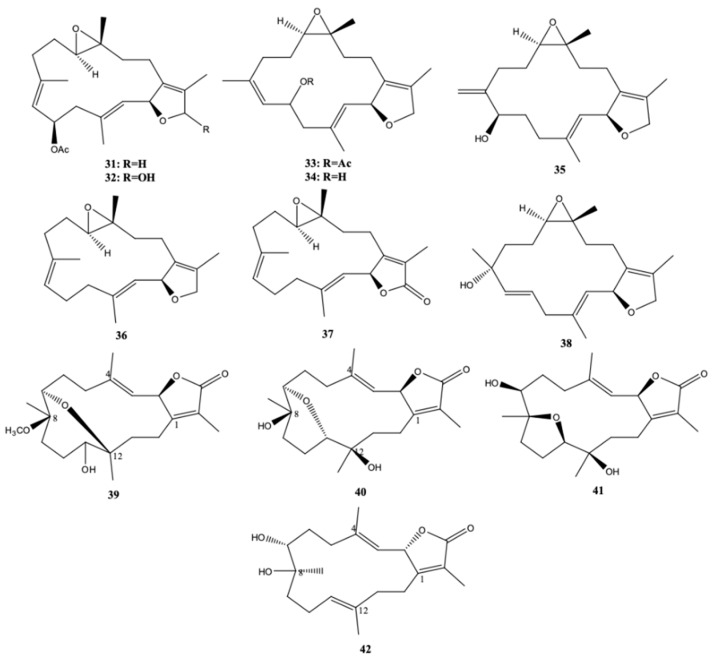
Cembranoid diterpenes isolated from *S. ehrenbergi*, from the Red Sea and South China Sea [20,27].

The chemical composition and biological properties of *S. ehrenbergi* from the South China Sea were studied by [27]. This study led to the isolation and identification of eight cembrane diterpenoids, including the five new sarcophytonoxides A–E (**31**–**35**), and three known ones, (2S,11R,12R)-isosarcophytoxide (**36**), (+)-isosarcophine (**37**), and 8-hydroxyisosarcophytoxide-6-ene (**38**) (Figure 5). All cembranoids were inactive (IC_50_ > 25 μM) against the human ovarian cancer cell line A2780 [27].

Sarcoehrenbergilid A–C (**39**–**41**), three new cembane diterpenoids, along with two known cembrane diterpenoids, sarcophine (**17**), (+)-7α,8β-dihydroxydeepoxysarcophine (**42**) (Figure 5), among other terpenoids, were isolated and characterized from the Red Sea soft coral *S. ehrenbergi* [20]. Cytotoxic activity of cembrane diterpenoids was performed using three human tumor cell lines (lung or A549; colon or Caco-2; and liver or HepG2). The compounds (**39**), (**41**), (**42**), and the non-cembrane diterpenoids sinulolide A and sinulolide B were moderately active against A549 and HepG2, with IC_50_ = 43.6 − 98.6 μM [20].

**Figure 6:**
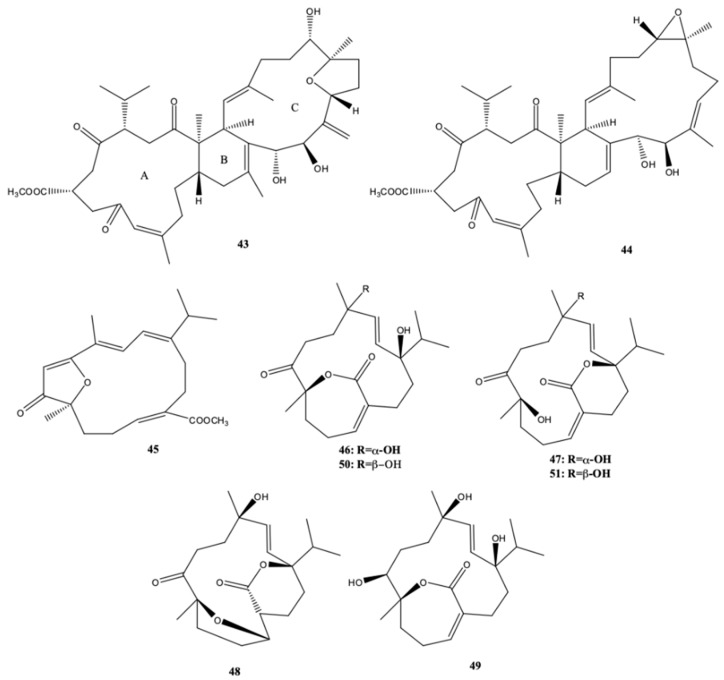
Biscembranoids and cembranoid diterpenes isolated from *Sarcophyton elegans*, collected at Xisha Islands in the South China Sea [22].

From the South China Sea coral *S. elegans*, Li et al. [22] isolated two novel biscembranoids, sarelengans A (**43**) and B (**44**), five new cembranoids, sarelengans C–G (**45**–**49**) (Figure 6), along with the two known cembranoids sartrolide E (**50**) and sarcophelegan B (**51**). The two novel biscembranoids had a *trans*-fused A/B-ring conjunction between the two cembranoid unities, in contrast to all biscembranoids. Such finding led the authors to hypothesize an unusual biosynthetic pathway of these compounds [22]. Sarelengans B (**44**) and C (**45**) had moderate inhibitory activity against the lipopolysaccharide (LPS)-induced nitric oxide (NO) production in RAW264.7 macrophages. Their half maximal inhibitory concentration (IC_50_) values were 18.2 and 32.5 μM, respectively.

**Figure 7:**
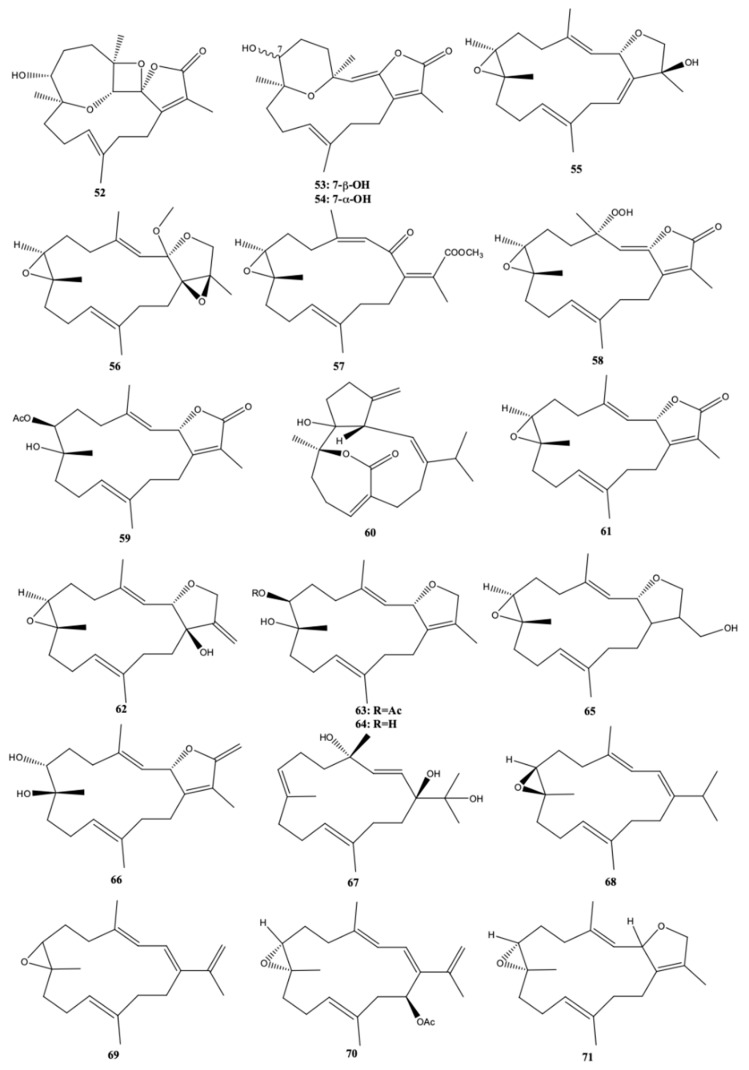
Cembranoid diterpenes isolated from *S. stellatum*, from the coast of Dongsha Atoll, Taiwan [33] and coast of Madagascar [24].

From the ethyl acetate extract of *S. stellatum*, collected along the Coast of Dongsha Atoll, Taiwan, in the north of the South China Sea, Ahmed et al. [33] isolated and identified six the new polyoxygenated cembrane-based diterpenoids stellatumolides A-C (**52**–**54**), stellatumonins A (**55**) and B (**56**), and stellatumonone (**57**) (Figure 7) together with two known related cembrane compounds but isolated for the first time from a natural source [hydroperoxyde obtained by autoxidation of dihydrofuranocembranoid (**58**) and 7β-acetoxy-8α-hydroxydeepoxy-sarcophyne (**59**)], and eight known related compounds [sarsolilide (**60**), (+)-sarcophine (**61**), laevigatol (**62**), sarcophytonin E (**63**), sarcophytonin C (**64**), 17-hydroxycarcophytoxide (**65**), 7β,8α-dihydroxydeepoxy-*ent*-sarcophine (**66**), and crassumol A (**67**)]. Only (**61**) showed anti-inflammatory activity by reducing the expression of cyclooxygenase-2 (COX-2) at 25–100 μM, and inducible nitric oxide synthase (iNOS) in LPS-stimulated RAW264.7 cells, at 50 and 100 μM. (+)-Sarcophine (**61**) were even better nonselective COX-2 inhibitor than ibuprofen and aspirin, but less effective than the selective COX-2 inhibitor celecoxib [33]. The compounds isolated were not cytotoxic against the human hepatocellular liver carcinoma (HepG2), human breast cancer (MDA-MB231), and human lung adenocarcinoma (A549) cell lines (IC_50_ > 20 μg/mL) [33].

Rahelivao et al. [24] investigated three soft corals (*S. stellatum, Capnella fungiformis* and *Lobophytum crassum*) and the sponge *Pseudoceratina arabica* from the coast of Madagascar (Indian Ocean). Concerning *S. stellatum*, the authors reported a new (+)-enantiomer of the cembranoid (1*E*, 3*E*)-7,8-epoxycembra-1,3,11,15-tetraene (**68**) produced by this organism. More three cembranoids were isolated and identified, by the authors, from *S. stellatum*: (+)-(7*S*,8*S*)-epoxy-7,8-dihydrocembrene C (**69**), (+)-(7*R*,8*R*,14*S*,1*Z*,3*E*,11*E*)-14-acetoxy-7,8- epoxycembra-1,3,11-triene (**70**), and (−)-(2*R*,7*R*,8*R*)-sarcophytoxide (**71**) (Figure 7). The biological properties, particularly antiplasmodial activity against the FCM29 strains of *Plasmodium falciparum* and antimicrobial was only studied with some extracts of the sponge *Pseudoceratina arabica*. Only the methanolic extract of *S. stellatum*, was biologically evaluated against *P. falciparum*. It presented only a moderate inhibition activity (IC_50_ = 35.20 μg/mL) [24].

**Figure 8:**
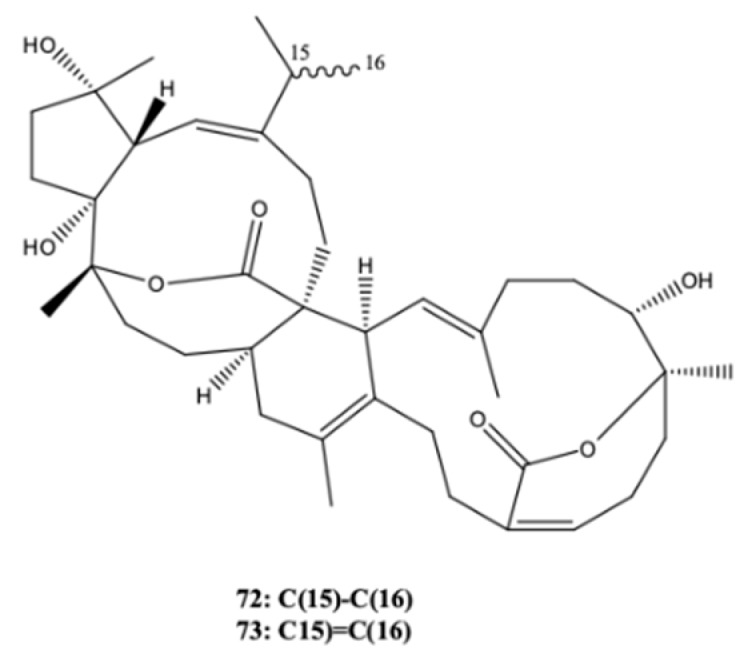
Biscembranoid-like compounds, bissubvilides A (**72**) and B (**73**) from the soft coral *Sarcophyton subviride*.

Two new biscembranoid-like compounds were obtained from the soft coral *Sarcophyton subviride* from the coast of Xisha, Hainan Province (China) [26]. They were bissubvilides A (**72**) and B (**73**) (Figure 8), that resulted from a Diels-Alder cycloaddition of two cembrane monomers. These compounds did not present any cytotoxic activity against human osteosarcoma MG-63 (IC_50_ > 30 μM) or A549 lung cancer (IC_50_ > 25 μM) cells or Huh7 human hepatology cancer stem cells (IC_50_ > 50 μM). Sarsolilide (**60**) was also detected in the soft coral *Sarcophyton subviride* from the coast of Xisha [26].

**Figure 9:**
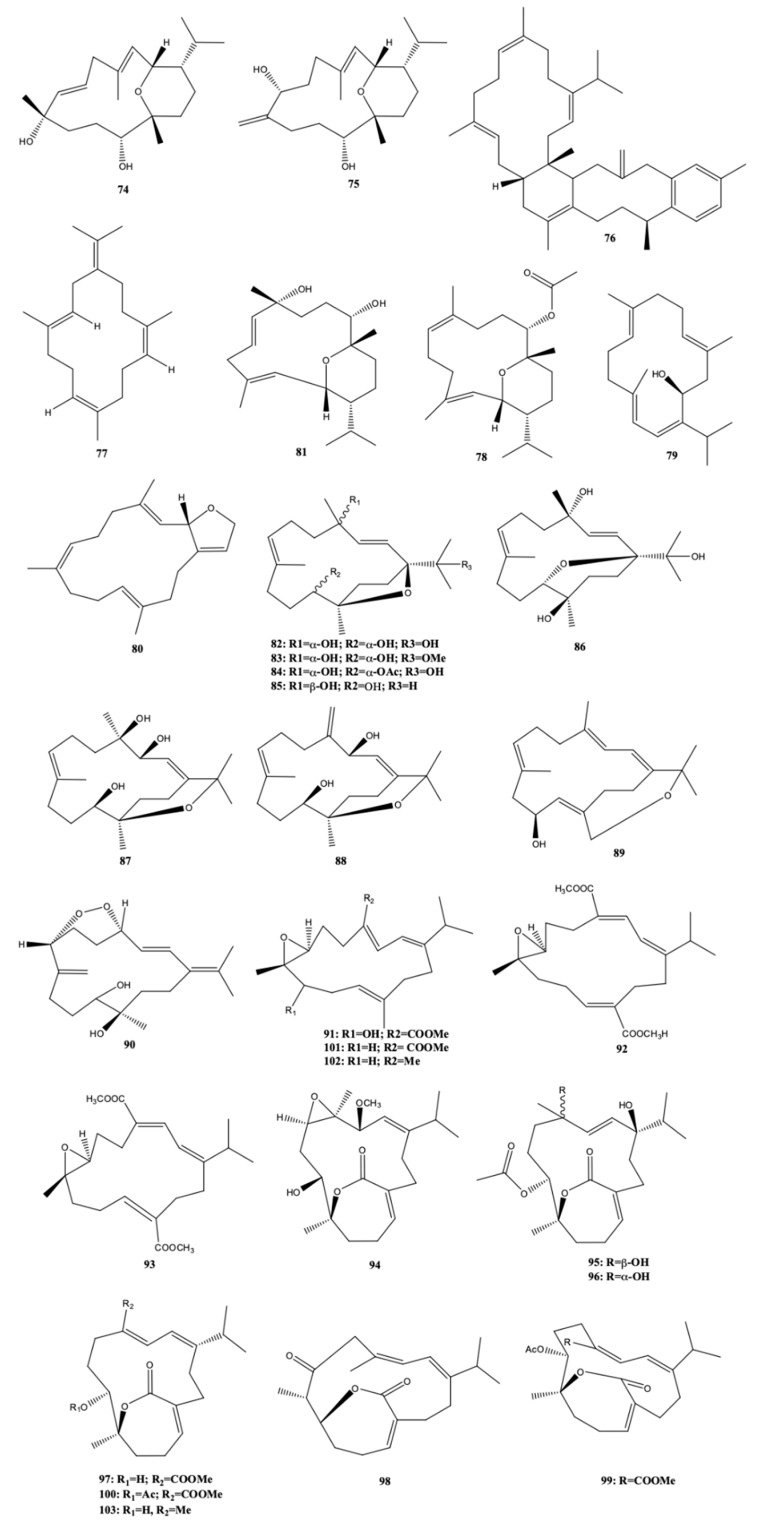
Cembranoid diterpenes isolated from *S. trocheliophorum*, from the Red Sea and South China Sea [23,25,28,34].

The antimicrobial activity of two new cembranoid diterpenes [sarcotrocheldiol A (**74**) and B (**75**)] and one new tetracyclic biscembrane hydrocarbon [trocheliane (**76**)] (Figure 9) isolated from the Red Sea soft coral *Sarcophyton trocheliophorum* was evaluated by Zubair et al. [28]. Along with this new compounds, the known diterpene cembrene C (**77**) was also isolated and identified from the same natural source. Trocheliane (**76**) was active against the two multidrug-resistant bacteria *Acinobacter baumannii* and *Staphylococcus aureus*. The MIC of this compound ranged from 4 to 6 μM for all the tested bacteria (*A. baumannii, S. aureus, S. epidermidis, Streptococcus pneumoniae, Escherichia coli, Klebsiella pneumonia*, and *Pseudomonas aeruginosa*) [28].

Along with the known compounds sarcotrocheliol acetate (**78**), (+)-sarcophytol A (**79**), and (−)-sarcophytonin A (**80**) (Figure 9), Shaaban et al. [25] isolated and identified 9-hydroxy-10,11-dehydro-sarcotrocheliol (**81**), a new pyrane-based cembranoid diterpene, from the organic extract of the Red Sea soft coral *S. trocheliophorum*. All compounds isolated by the authors from the soft coral *S. trocheliophorum* did not possess any antimicrobial activity towards *Bacillus subtilis, S. aureus, Streptomyces viridochromogenes* (Tü 57), *Escherichia coli, Candida albicans, Mucor miehei, Chlorella vulgaris, Chlorella sorokiniana, Scenedesmus subspicatus, Rhizoctonia solani*, and *Pythium ultimum*, at 40 μg per disk. The cytotoxicity of the four compounds against brine shrimp was also absent [25].

Nine new cembranoids, sarcophytrols M–U (**82**–**90**) (Figure 9), were isolated from the South China Sea soft coral *S. trocheliophorum*, along with one already known. Such new compounds possess diverse types of cyclized rings: furan rings in sarcophytrols M-P (**82**–**85**), pyran rings in sarcophytrols, oxepane, and peroxyl rings in sarcophytrols T (**89**) and U (**90**), respectively. Sarcophytrols R (**87**) and S (**88**) had a rare bicyclic skeleton of the decaryiol-type, as reported for the first time for the same genus of soft coral *S. decaryi* [23]. The bioassay for evaluating the capacity for inhibiting human protein tyrosinase phosphatase 1B (PTP1B) enzyme, important for the treatment of type-2 diabetes and obesity, all compounds isolated from the soft coral *S. trocheliophorum* did not provide positive results. Cytotoxicity against the human tumor cell lines HL-60 (Human promyelocytic leukemia cells) and K-562 (human erythroleukemia cells), as well as the antibacterial activity of the same compounds against *P. aeruginosa* also revealed negative [23]. Later on, the authors isolated and identified highly oxidative new cembranoids with dienoate moieties [sarcophytonolides S-U (**91**–**93**)] or an α,β-unsaturated ε-lactone [sartrolides H-J (**94**–**96**)] (Figure 9) from the soft coral *S. trocheliophorum* collected in the same region (Yalong Bay, Hainan Province, South China Sea), along with seven known related analogues [deacetylemblide (**97**), 4*Z*,12*Z*,14*E*-sarcophytolide (**98**), sarcrassin D (**99**), emblide (**100**), sarcophytonolide A (**101**), (*E*,*E*,*E*)-7,8-epoxy-l-isopropyl-4,8,12-trimethylcyclotetradeca -l,3,11-triene (**102**), and (4*Z*,8*S*,9*R*,12*E*,14*E*)-9-hydroxy-1-isopropyl-8,12-dimethyl-oxabicyclo [9.3.2]- hexadeca-4,12,14-trien-18-one (**103**)] [34]. Sartrolide H (**94**) and 4*Z*,12*Z*,14*E*-sarcophytolide (**98**) had moderate inhibitory activity against protein tyrosine phosphatase 1B (key target for the treatment of type-II diabetes and obesity) with IC_50_ = 19.9 and 15.4 μM, respectively, significantly less than the positive control, oleanolic acid (IC_50_ = 2.6 μM). 4*Z*,12*Z*,14*E*-Sarcophytolide (**98**) had also moderate inhibitory activity against *Staphylococcus aureus* Newman strain (MIC_50_ = 250 μM), less than the positive control, fosfomycin (MIC_50_ = 137.4 μM) [34].

**Figure 10:**
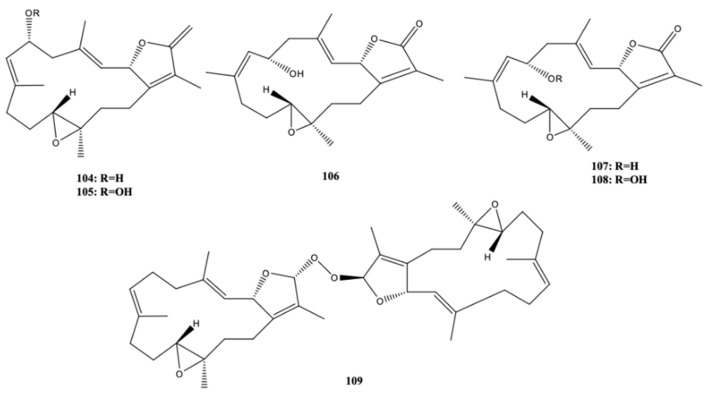
New cembranoid diterpene isolated from *S. cherbonnieri* from the Jihui Fish Port, Taiwan (Philippine Sea) [35].

Six new cembranoids, cherbonolides A–E (**104**–**108**) and bischerbolide peroxide (**109**) (Figure 10) were isolated from the Formosan soft coral *S. cherbonnieri*, along with one known cembranoid, isosarcophine (**37**) (Figure 5). All compounds exhibited the capacity for inhibiting the production of superoxide anions and elastase release in *N*-formyl-methionyl-leucyl-phenylalanine/cytochalasin B (fMLF/CB)-induced human neutrophils, that is, they possessed anti-inflammatory activity. Bischerbolide peroxide (**109**) exhibited the highest capacity for inhibiting the generation of superoxide anions (IC_50_ = 26.2 μM), but moderate activity on elastase release at the same concentration along with **104** and **106**, at 30 μM [35].

## 4. New Cembrane Derivatives from the Genus *Sinularia*

Soft coral *Sinularia* consists of more than 150 species [36]. As aforementioned for *Sarcophyton* sp., reviews have also been made regarding the discovery of new compounds and their biological activities up to 2016 [11,12,13,14,17]. During the period 2016-2017, only three publications could be found in the *Web of Science* utilizing the words *cembrane, cembranoid,* and *Sinularia* (one study in 2016 [37] and two studies in 2017 [38,39]), whereas in the first half of 2018 seven publications could be found using the same database [40,41,42,43,44,45,46]. The species reported in eight works included one *S. erecta,* one *S. compacta*, two *Sinularia* sp., and four *S. flexilibis*, all of them from the South China Sea (Table 3). In addition, there are other works in which one of them was focused on the biological activity of sinularin extracted from marine soft corals (*S. flexibilis* and *S. manaarensis*) [40] and the other one identified five cembranoid diterpenes (isosinulaflexiolide K, sinulaflexiolide K, sandensolide, sinularin, and dendronpholide F) obtained from cultured soft coral *S. flexibilis* [41] but without any biological activity determination. The anti-breast cancer activity (SKBR3 and MDA-MB-231 cells) of sinularin extracted from marine soft corals (*S. flexibilis* and *S. manaarensis*) were detected by [40], nevertheless, it was almost non-toxic against breast normal (M10) cells, at least after 24 h treatment. On the SKBR3 cells, the mechanisms involved on the anticancer activity included the induction of the G2/M cycle arrest, apoptosis of cells, and oxidative stress and DNA damage, as well as the pancaspase activity, and activation of poly(ADP-ribose) polymerase (PARP), and caspases 3, 8, and 9 [40].

**Figure 11:**
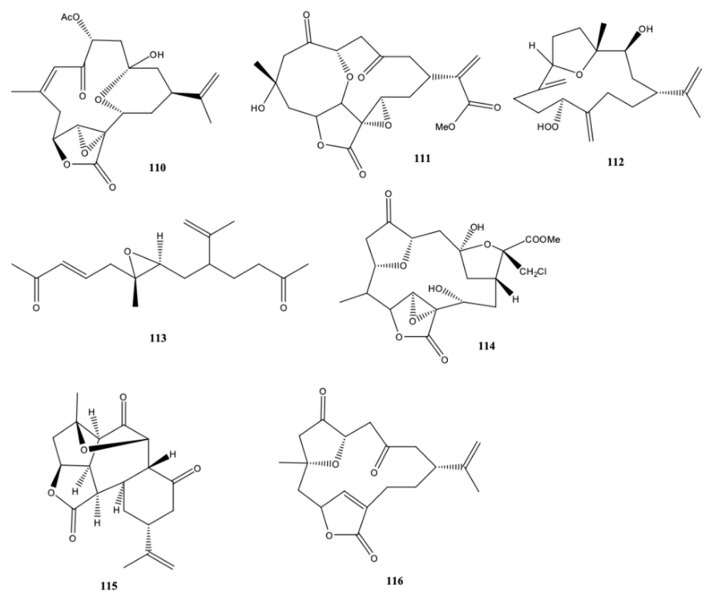
Cembranoid diterpene derivatives isolated from *Sinularia erecta* from South China Sea [37].

Not only does the sinularin have anticancer activity; but also species of the genus *Sinularia* are rich in bioactive cembranoids and norcembranoids [37]. These authors reported for the first time two new norcembranoids [sinulerectol A **(110**) and B (**111**)] (Figure 11), a new cembranoid [sinulerectol C (**112**)] and a new degraded cembranoid sinulerectadione (**113**), alongside some known isoprenoids [norcembrene, sinularectin (**114**) and ineleganolide (**115**)] and an unnamed norcembrene (**116**) isolated from an extract of the marine soft coral *Sinularia erecta* from South China Sea (off the coast of Dongsha Atoll) [37]. Sinulerectadione (**113**) exhibited inhibitory activity against myelogenous leukemia (K-562) and acute lymphoblastic leukemia (MOLT-4) cell lines with IC_50_ values of 8.6 and 9.7 μM, respectively, whereas sinulerectol C (**112**) was effective against MOLT-4 cell lines (9.2 μM). The anti-inflammatory activity of **110** and **111** on neutrophil pro-inflammatory responses was potent when evaluated by measuring the capacity for suppressing formyl-Met-Leu-Phe/cytochalasin B (fMLP/CB)-induced superoxide anion generation (IC_50_ = 0.9 and 3.8 μM, respectively) and elastase release in human neutrophils (113 and 93% inhibition at the same concentration, respectively) [37].

**Figure 12:**
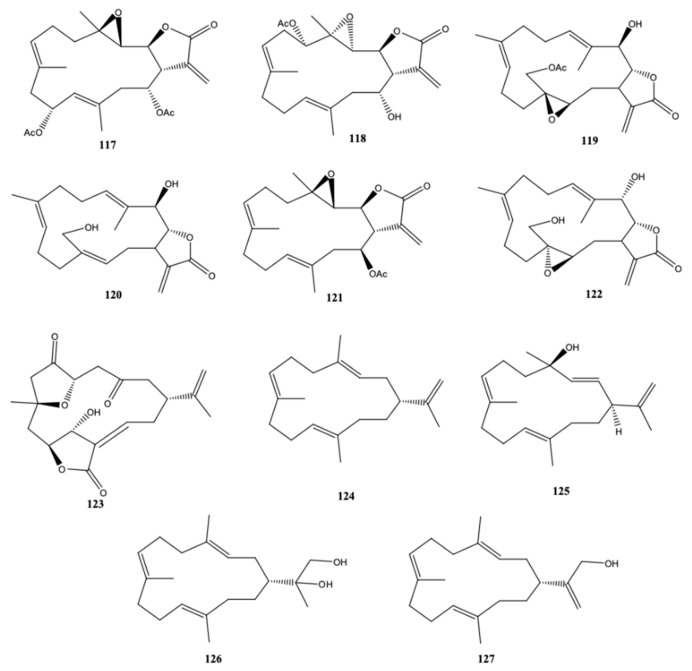
Cembranoid diterpene derivatives isolated from *Sinularia compacta* from the South China Sea (Tongguling National Nature Reserve of Coral Reefs) [38].

In the first report about chemical constituents of *S. compacta*, Wang et al. [38] reported three new compounds in the genus *Sinularia* [lobomichaolide (**117**), michaolide F (**118**), and 20-acetylsinularolide B (**119**)], along with more eight compounds already found in the same genus of soft coral [presinularolide B (**120**), 14-acetoxy-3,4-epoxycembra-7,11,15-trien-17,2-olide (**121**), sinularolide C (**122**), 5-epi-sinuleptolide (**123**), 1-isopropyl-4,8,12-trimethyl-cyclotetradeca-2,4,7,11- -tetraene (**124**), (1*R*,4*R*,2*E*,7*E*,11*E*)-cembra-2,7,11-trien-4-ol (**125**), sinulariol B (**126**), and sinulariol D (**127**)] (Figure 12), all of them are 14-membred cembranoid diterpenes. The first six cembranoid diterpenes possess a α-methylene-γ-lactone moiety, make them cytotoxic, as well as anti-HIV and antituberculosis [43]. However, only 5-epi-sinuleptolide (**123**) exhibited cytotoxic activity against the tumor cell lines HCT-116 and A-549 (IC_50_ values of 10.1 and 14.7 μM, respectively). Michaolide (**119**) and 20-acetylsinularolide (**119**) were lethal toward brine shrimp *Artemia salina* with lethal ratios of 90.5% and 90.0%, respectively, at a concentration of 50 μg/mL [38].

**Figure 13:**
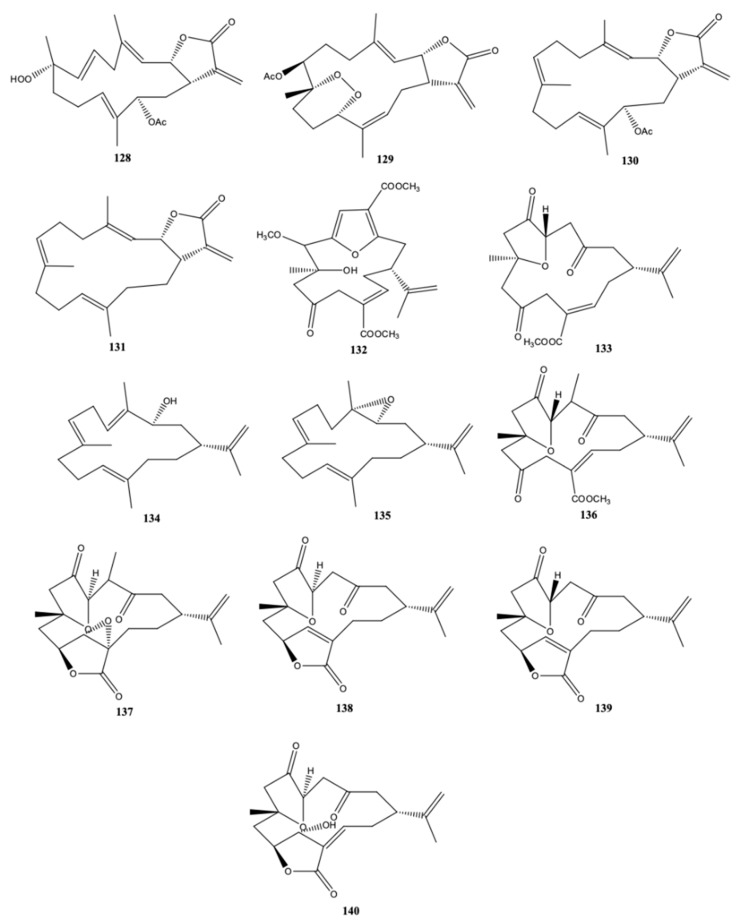
Cembranoid diterpene isolated from *Sinularia* sp. collected in Mantanani Island, Sabah and Yongxing Island, both in the South China Sea [42,43].

Sinularolide F (**128**) (Figure 13), a new cembranoid, along with the known denticulatolide (**129**), isolated from the Bornean *Sinularia* sp. (Mantanani Island, Sabah) also exhibited anticancer activity against HL60 cell lines (human pro-myelocytic leukemia) by triggering apoptosis [42]. The possible mechanisms involved include the upregulation of Bax (bcl-2-like protein 4), the down regulation of Bcl-xL (B-cell lymphoma-extra-large) and the activation of caspase-3 [42]. Sinularolide F (**128**) and denticulatolide (**129**) also had anti-inflammatory activity by inhibiting NO, IL-1β and IL-6. According to the authors the activities found could be attributed to the β-configuration of methyl group at C-8 as well as to the presence of hydroperoxy or peroxy groups also bound to C-8 [42]. Cembranolide (**130**), (*E*,*E*,*E*)-6,10,14-trimethyl-3-methylene-*cis*-3,4,5,8,9,12,13,15-octahydrocyclotetradeca[β]furan-2(3*H*)-one (**131**).

Qin et al. [43] identified new sesquiterpenoids and cembranoids in the Xisha soft coral *Sinularia* sp. Sinulins C (**132**) and D (**133**) (Figure 13) were the new cembranoids isolated and identified by the authors. Of the new cembranoids, only sinulin D (**133**) had mild inhibitory activity against PTP1B (protein tyrosine phosphatase 1B), nevertheless the known cembranoid 5-episinuleptolide (**123**) (Figure 12) showed activity against HeLa (human cervical epitheloid carcinoma) and HCT-116 (human colon carcinoma) cell lines [43]. Seven known cembranoid diterpenes were also detected in the Xisha soft coral *Sinularia* sp.: (1*R*,3*S*)-cembra-4,7,11,15-tetraen-3-ol (**134**), (1*R*,3*S*,4*S*,7*E*,11*E*)-3,4-epoxycembra-7,11,15-triene (**135**), norcembrene 5 (**136**), norcembrenolide C (**137**), sinularcasbane O (**138**), scabrolide F (**139**), and sinuleptolide (**140**).

**Figure 14:**
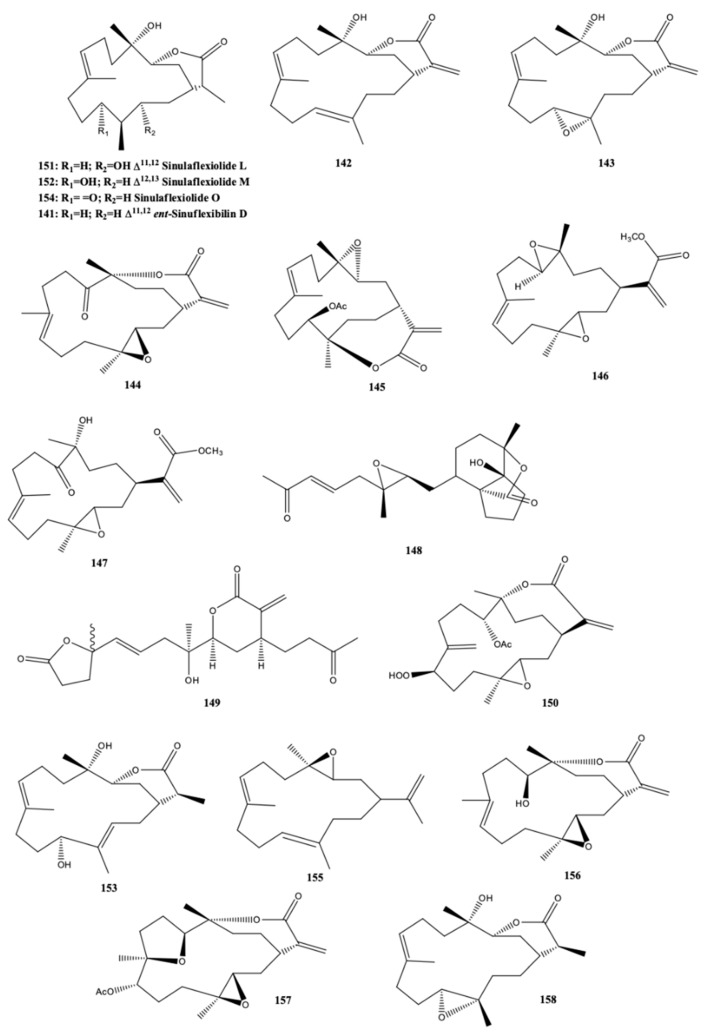
Cembranoid diterpene-derivarives from *Sinularia flexibilis* from the South China Sea (Sanya Bay, Hainan Island; Mantanani Island, Sabah; Megalum Island, Sabah; Yongxing Island, Coast of Liuqiu, Taiwan) [39,44,45,46]. 5-Dehydrosinulariolide and 11-dehydrosinulariolide have the same number, because the chemical structure found in the references is the same for both names.

*ent*-Sinuflexibilin D (**141**) (Figure 14) was a new cembranoid isolated by [44] from a population of Bornean soft coral *Sinularia flexibilis*, along with seven known compounds, including a sesquirerpene (muurolene) only detected until now in *Cistus ladanifer*. The new cembranoid together with the known cembranoid diterpenes [14-deoxycrassin (**142**), sinularin (**143**), 5-dehydrosinulariolide (**144**) and 11-*epi*-sinulariolide acetate (**145**)] exhibited activity against adult T-cell leukemia (ATL). In addition, (**141**) and the remaining cembranoid diterpenes were also able to act against the growth of three strains of marine fungi *Exophiala* sp. NJM 1551, *Lagenidium thermophilum* IPMB 1401 and *Haliphthoros sabahensis* IPMB 1402. These microorganisms cause infection in fishes and mangrove crabs, being *H. sabahensis* a new fungal species described very recently [44].

Cytotoxicity activity of cembranoids isolated from the Taiwanese soft coral *S. flexibilis* was evaluated by Wu et al. [45]. According to the authors five new cembranoid-related diterpenes [flexibilisins D (**146**) and E (**147**), secoflexibilisolides A (**148**) and B (**149**), and flexibilisolide H (**150**)] (Figure 14) were isolated from this soft coral along with nine known compounds. The known 11-dehydrosinulariolide (**144**) possessed selective cytotoxicity towards P388 (murine leukemia) cell line, and (**145**) presented remarkable cytotoxicity activity and selectivity on P-388 and HT-29 (human colon carcinoma) cell lines [45]. The new compounds, however, did not exhibit any activity towards these two types of cells since they presented IC_50_ values over than 40 μM. The anti-inflammatory activity was also determined but only (**142**) had capacity for inhibiting superoxide anion formation and elastase release in *N*-formyl-methionyl-leucyl-phenylalanine/cytochalasin B (fMLF/CB)-induced human neutrophils at a concentration of 10 μM [45]. The anti-inflammatory activity of the cembranoids isolated from the *S. flexibilis* collected off the coast of Yalong bay, Sanya in Hainan province (China) was also evaluated by Zhao et al. [46] through the capacity for inhibiting the LPS-induced NO and TNF-α generation in the RAW264.7 macrophage cells. Sinularin (**143**) was the best among the compounds isolated by the authors, displaying inhibition percentages higher than 80%, at 10 μM, significantly higher when compared to the new cembranoid diterpenes [sinulaflexiolides L-O (**151**–**154**) and *ent*-sinuflexibilin D (**141**)] identified by the authors [46].

Seven cembrane diterpenes were isolated from the soft coral of *S. flexibilis* from China (Sanya Bay, Hainan Island) [epoxycembrane A (**155**), sinularin (**143**), sinulariolide (**156**), (1*R*,13*S*,12*S*,9*S*,8*R*,5*S*,4*R*)-9-acetoxy-5,8:12,13-diepoxycembr-15(17)-en-16,4-olide (**157**), 11-dehydrosinulariolide (**144**), (-)14-deoxycrassin (**142**), and dihydrosinularin (**158**)] (Figure 14). Epoxycembrane A (**155**) was for the first time reported in *S. flexibilis* [39]. Tributyltin and copper are antifouling largely used in order to deter marine fouling organisms on the surfaces of artificial structures submerged in the sea, but they present some drawbacks particularly due to their adverse environmental impacts [39]. For this reason, several attempts have been made for finding more environmental friendly compounds. Wang et al. [39] assayed the antifouling activity on the larvae of the bryozoan *Bugula neritina* and the barnacle *Balanus albicostatus* of all the cembranoid diterpenes isolated from *S. flexibilis*. With the exception of (**143**), all remaining ones presented activity, and particularly (**142**) had the highest antifouling activity against both *Bugula neritina* and barnacle *Balanus albicostatus* [the concentrations of the compound that inhibited settlement by 50% relative to the control (EC_50_) were 3.90 μg/mL and 21.26 μg/mL, respectively], and low toxicity against *B. albicostatus* larvae [the concentration that originates 50% mortality) (LC_50_) > 100 μg/mL]. According to the authors [39], the antifouling activity of (**142**), (**144**), (**155**), (**157**), and (**158**) was reported for the first time.

## 5. New Cembrane Derivatives from the Genus *Lobophytum*

The genus *Lobophytum* is rich in cembranoids and more than 250 different structures had been isolated from the genus *Lobophytum* [9]. The number of publications about the structure of new cembranoid diterpenes and/or their biological properties during the period 2016-2018, is as follows: in 2014, three publication in *Web of Science*, using the terms *cembrane, cembranoid,* and *Lobophytum* could be found [46,47,48], as well as in 2015 [49,50,51]), and 2016 [52,53,54]. In 2017, the number of publications was 5 [9,24,55,56,60], whereas until August 2018, only one work was found [57].

**Figure 15:**
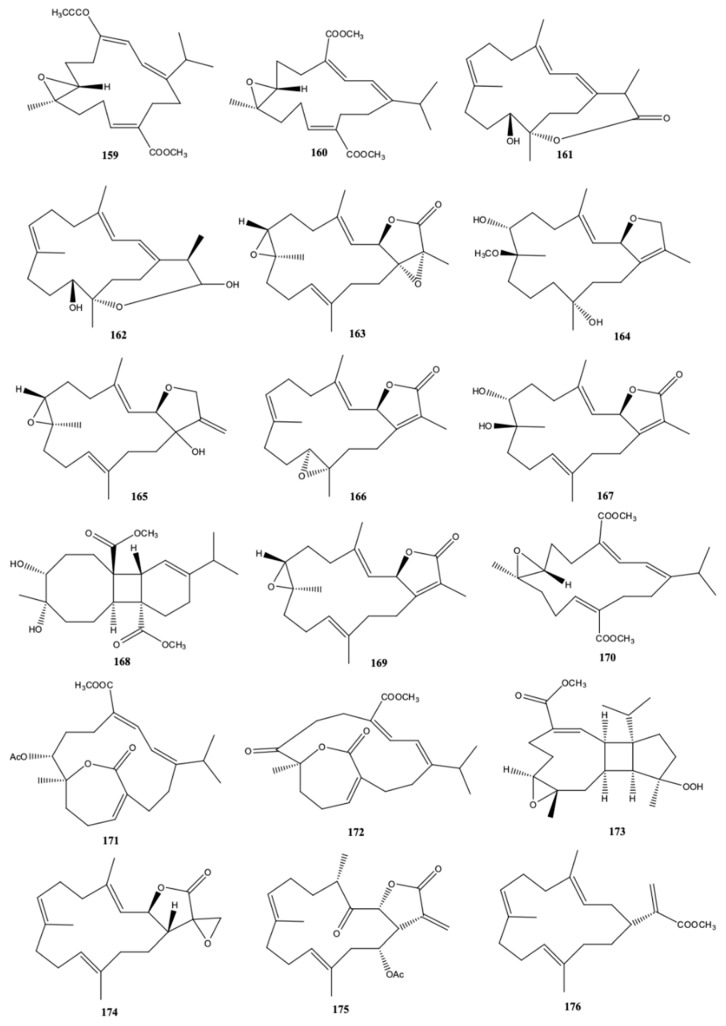

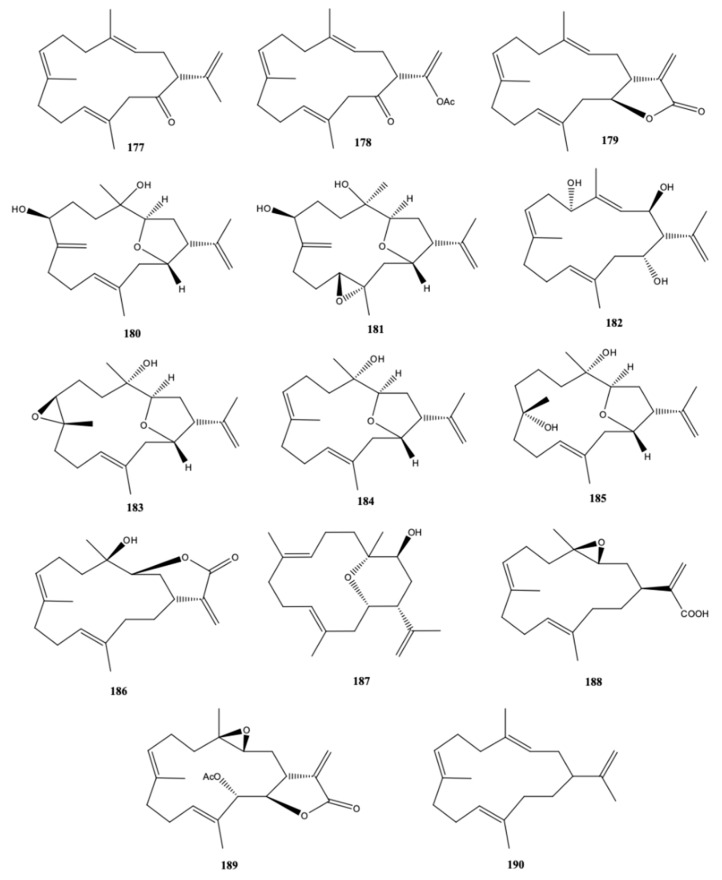
Cembranoid diterpenes from wild *Lobophytum crassum* from China (South China Sea and Twain) [9,54,55,56] and from aquaculture *L. crassum* [57].

During 2016–2018, the most studied of soft coral species was *L. crassum*, either in terms of biological properties of known cembranoids or research of new cembranoid diterpenes. This species was collected in several places (Table 4). Species *L. crassum* is well known to produce oxygenated cembranoids. The structural variety of these metabolites is often correlated with geographic variation and environmental conditions [54]. The soft coral *L. crassum* from the South China Sea was studied by [54] and from this study, the authors isolated and identified nine new cembranoids [locrassumin A (**159**), B (**160**), D-G (**161**-**164**), (-)-laevigatol B (**165**), (-)-isosarcophine (**166**), and (–)-7*R*,8*S*-dihydroxydeepoxysarcophytoxide] (**167**), a diterpene possessing a tetradecahydrobenzo [3,4] cyclobuta [1,2,8] annulene ring system (locrassumin C) (**168**), and eight known cembranoids [(-)-sarcophytoxide (**18**), *ent*-sarcophine (**169**), sarcophytonolide O (**170**), sartrolide G (**171**), emblide (**100**), sarcrassin D (**99**), ketoemblide (**172**), and methyl sarcotroate B (**173**)] (Figure 15). The anti-inflammatory activity of all compounds was evaluated, after measuring the lipopolysaccharide (LPS)-induced NO (nitric oxide) production in mouse peritoneal macrophages. Compounds (**159**), (**161**), (**169**), (**170**), and (**172**) exhibited moderate inhibition against LPS-induced NO production with IC_50_ values of 8–24 μM (Table 4). The remaining metabolites did not present inhibitory effect (IC_50_ > 30 μM) [54].

The wild soft coral of *L. crassum*, collected around 8 m off the coast of Pingtung, Taiwan, was isolated and identified two new compounds [lobophyolide A (**174**) and B (**175**)], and the known cembranoid diterpenes [16-methoxycarbonyl-cembrene A (**176**), sinarone (**177**), sinulariol D (**127**), 16-acetyl-sinulariol D (**178**), and (*E,E,E*)-6,10,14-trimethy-3-methylene-*trans*-3a,4,7,8,11,12,15,15*a*-octahydrocy clotetradeca[β]furan-2(3H)-one) (**179**) (Figure 15) [9]. The anti-inflammatory activity of the cembranoid compounds was evaluated studying the effect of these compounds on the LPS-induced interleukin 12 (IL-12) release and NO production in dendritic cells [9]. The results showed that (**174**), (**176**) and sinulariol D (**127**) (<50 μg/mL) presented a potent inhibitory effect of IL-12 and NO release (86.1–96.2%). Moreover, the same compounds also had considerable cytotoxicity (Table 4) [9].

Mohamed et al. [56] isolated from *L. crassum*, collected off the coast of Dongsha Atoll (South China Sea), three new cembranoids [lobophylins F-H (**180**–**182**)], together with three known ones lobophylin C (**183**–**185**) (Figure 15). Rahelivao et al. [24] did not isolate cembranoids from the soft coral *L. crassum* extract from the coast of Madagascar, but only they reported the moderate activity of the crude methanol extract against the malarial parasite FCM29 strain of *Plasmodium falciparum* (IC_50_ value of 33.15 μg/mL). In other work, Lin et al. [55] studied the anticancer ability of lobocrassin B (**186**) (Figure 15), a natural cembrane diterpenoid previously isolated from the soft coral *L. crassum*. The authors reported that this compound exerted cytotoxic effects for concentrations <10 μM on lung cancer CL-15 and H520 cells lines, not only by decreasing cell viability but also by inducing apoptosis, oxidative stress and mitochondrial dysfunction (increased level of Bax, cleaved caspase-3, -9 and -8, and suppression of Bcl-2). Nevertheless, much higher concentration was necessary to add to the normal human bronchial epithelium (BEAS-2B) for exerting cytotoxic effect (>25–50 μM), which means that (**186**) preferably causes cell death of carcinogenic cells than normal cells.

Recently, Peng et al. [57] studied the chemical composition and biological activity of cembranoid diterpenes isolated from aquaculture soft coral *L. crassum* and compared the results with those of wild type. Two new cembrane-based diterpenoids [culobophylins D (**187**) and E (**188**)] (Figure 15) were identified in the aquaculture soft coral together with ten known cembranoids. The known 13-acetoxysarcocrassocolide (**189**), lobocrassin B (**186**) and 14-deoxycrassin (**142**) were the most active compounds against diverse leukemia cell lines (K562, U937, Molt4, and Sup-T1). These results may reveal that the presence of α-methylene-γ-lactone or α-methylene-δ-lactone moieties in the cembranoid diterpenes is important for the cytotoxic activities found by the authors [57].

Al-Footy et al. [52] isolated diverse secondary metabolites (sesquiterpenes, steroid type compounds and only one known cembrane diterpene) from the soft coral *Lobophytum* sp. were collected off the Red Sea Coast, in Jeddah, Saudi Arabia. The isolated cembrane diterpene was cembrene A (**190**). This cembrane diterpene showed moderate antibacterial activity against *Acinetobacter* sp., *E.coli, Klebsiella pneumonia, Pseudomonas aeruginosa, Staphylococcus aureus, Staphylococcus epidermidis, Streptococcus pneumonia*. But it presented high toxicity against brine shrimp *A. salina* and antitumor activity against Erhlich carcinoma cells with median lethal dose (LD_50_) values of 25 and 50 μg/mL, respectively.

**Figure 16:**
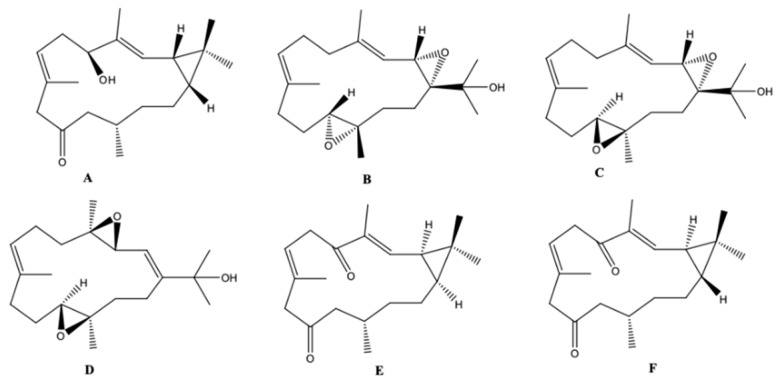
Casbanes and cembrane diterpenes from a soft coral *Lobophytum* collected in the coast of Irabu Island (Okinawa, Japan) [53].

Roy et al. [53] isolated and identified 7 cembrane-type diterpenes from the coast of Irabu Island (Okinawa, Japan), a soft coral *Lobophytum* sp.: a new rare casbane-tipe diterpenoid **A**, two new cembrane diterpenoids (**B** and **C**); and four known cembrane diterpenoids (**D**–**G**). The authors did not attribute names for the structures presented (Figure 16). The structures of the compounds were obtained by analysis of spectroscopic data, using IR, ^1^H-NMR and ^13^C-NMR, 1D and 2D-NMR measurements (COSY, HSQC, HMBC, and NOESY), and HRESIMS spectra, after extraction with acetone and fractionation by chromatographic processes. The authors reported that the compounds A–E showed weak anti-bacterial activity (*Staphylococcus aureus, Salmonella enterica* and *E. coli*). Compounds A–C showed moderate cytotoxicity against human colon cancer cells (HCT116) with IC_50_ values ranging from 135.57 to 177.11 μM, and anti-inflammatory activity in LPS/IFN-γ (LPS/interferon-γ)- -stimulated RAW 264.7 macrophages cells (IC_50_ 41.21–74.76 μM) [53].

## 6. Concluding Remarks

The use of increasingly sophisticated equipment has permitted to identify new compounds, including natural compounds of marine origin. The aim is to find remarkable biological properties for possible application in Medicine. A huge diversity of chemical structures have been isolated and evaluated in biological terms from marine organisms. The soft corals belonging to the family Alcyoniidae are not an exception. They are, indeed, the target of several studies in searching for new products with biological properties, particularly antimicrobial, anti-inflammatory, and anti-tumoral activities. Cembranoids from soft corals of the genera *Sinularia, Lobophytum*, and *Sarcophyton* are the most well studied secondary metabolites of the specimens belonging to these genera. The chemical diversity of cembranoid diterpenes is remarkable and evident from previous studies. Such diversity can be attributed to the differences in environmental conditions between the different localities, like: surface temperature, salinity, nutrient concentrations, and turbidity. However, in the present review it is important to emphasize the following points: in *Sarcophyton* species the new compounds isolated by the authors belonged predominantly and by descending order to: isopropyl cembranes, 5-membered lactones, biscembranoids, and furanocembranoids; in *Sinularia* species, the new cembrane compounds were predominantly lactones (5 or 6-membered ones) and isoprenyl cembranes; in the *Lobophytum* species there was no predominance of any type of cembrane diterpenoids. In this case, isopropyl, isopropenyl, isoprenyl acid, 5- and 7-membered lactones, furanocembranoid or even casbane types were detected without any predominant one.

The diversity of results in what concerns the biological properties reported in several works can be partly attributed to the variability of chemical structures found, even though the presence of some chemical groups or their arrangement in the cembranoid diterpene core can be determinant in such activities. In the present review, citotoxicity was evaluated in seven works using diverse tumor cell lines (lung, liver, colon, osteosarcoma, ovarian, breast, leukemia) the species of the genus *Sarcophyton*. However, moderate activity was detected in only two researches; mainly against liver, lung (*S. ehrenbergi* from the Red Sea Coast) and breast (*Sarcophyton* sp. from Celebes Sea). The antibacterial activity was the second most important activity scrutinized by the researchers, but the activity was only moderate to weak or even absent for the microorganisms used. For example, for *Staphylicoccus aureus*, the activity found ranged for inactive (pyrane-based cembranoid diterpene) to active against multidrug resistant strain (the biscembrane hydrocarbon trocheliane). Anti-inflammatory activity was positive in all assays independent on the methodology used: inhibition of production of NO, interleukins, prostaglandins, TNF-α by LPS-LPS-stimulated macrophage cells; inhibition of expression of COX-2 or inhibition of superoxide anions generation. In contrast, in *Sinularia* species, there is a species (*S. flexibilis*) in which no anti-inflammatory or antioxidant activity was found, whereas in other cases *S. flexibilis* presented an anti-inflammatory activity. The citotoxicity towards several tumor cell lines were detected either in species of the genus *Sinularia* or *Lobophytum*, but with diverse strengths.

Although such diversity of results can be attributed to the chemical groups and their arrangement in the core structure, it would be advisable to find other approaches to identify biological properties. Sometimes it is unclear the reasons that led the researchers to determine certain activities as well as the choice of some cells or parameters. Whether for plant kingdom, the ethnobotany or ethnopharmacology are strong tools for searching bioactive compounds, in the marine world cannot provide enough information, whereby other approaches must be taken into account. World data libraries compiling the results obtained so far, along with structure-based drug design methods (e.g., molecular docking, structure-based virtual screening and molecular dynamics) can predict in some extent possible activities and interactions with diverse targets, maybe more adequate and less expensive than the current way generally followed. The resistance of microorganisms to antibiotics is of great concern, therefore the bactericidal activity found for 16-hydroxycembra-1,3,7,11-tetraene, or the activity against the two multidrug resistant bacteria *Acinobacter baumannii* and *Staphylococcus aureus* of trocheliane should be deeply studied not only in the search of new compounds with similar structures or, even better, those molecules can serve as templates for the construction of new molecules after adequate modifications.

## Figures and Tables

**Table 1:** Classification of cembrane diterpenoids.

Type	Subtype	Examples	Source	Structures
Simple cembrane	Isopropyl cembranes	Sarcophytol M (**1**)	*Sarcophyton glaucum*	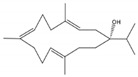
	Isopropenyl cembranes	Sinulariol C (**2**)	*Sinularia mayi*	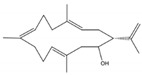
	Isopropyl/isopropenyl acid cembranes	Flexibilisin A (**3**)	*Sinularia flexibilis*	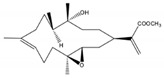
Cembranolides	5-Membered lactone	Deacetyldeepoxy lobolide (**4**)	*Lobophytum crassum*	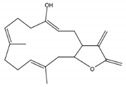
	6-Membered lactone	Manaarenolide A (**5**)	*Sinularia manaarensis*	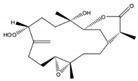
	7-Membered lactone	Sinuladiterpene (**6**)	*Sinularia flexibilis*	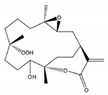
	8-Membered lactone	Echinodolide (**7**)	Brazilian medicinal plant *Echinodorus macrophyllus*	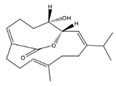
Furanocembranoids	-	Pukalide (**8**)	*Sinularia abrupt*	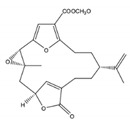
Biscembranoids	-	Lobophytone A (**9**)	*Lobophytum pauciflorum*	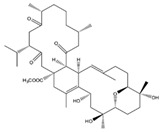
Special cembranes	Secocembranes	Mayolide A (**10**)	*Sinularia mayi*	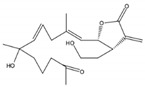
	13-Membered carbocyclic cembranoids	Sartol acetate (**11**)	Unidentified *Sarcophyton* species	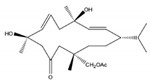
	Cembrane glycosides	Calyculaglycoside A (**12**)	Caribbean Gorgonian Octocoral *Eunicea* sp.	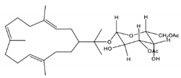
	Cembrane-africanane	Polymaxenolide (**13**)	*Sinularia maxima* x *S. polydactyla*	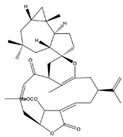
	Other cembranes	Planaxool (**14**)	Marine mollusk *Planair sulratru*	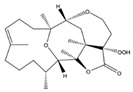

**Table 2:** Harvesting locations of the soft corals of the genus *Sarcophyton*, extraction solvent, new compounds identified and their biological properties.

Soft Coral	Extraction	New Bioactive Cembranoid Diterpene	Type	Biological Activities	Location	Reference
					**Red Sea Coast**	
*Sarcophyton trocheliophorum*	Acetone/room temperature	Trocheliane (**77**)	Biscembrane hydrocarbon	Activity against the two multidrug resistant bacteria *Acinobacter baumannii* and *Staphylococcus aureus* (MIC = 4.2 and 4.0 μM, respectively)	North of Jeddah, Saudi Arabia, Red Sea Coast (21°29′31′’N, 39°11’24′’E)	[28]
*Sarcophyton trocheliophorum*	Not reported	9-Hydroxy-10,11-dehydro-sarcotrocheliol (**82**)	Pyrane-based cembranoid diterpene	- Inactive against *Bacillus subtilis, Staphylococcus aureus, Streptomyces viridochromogenes* (Tü 57), *Escherichia coli, Candida albicans, Mucor miehei, Chlorella vulgaris, Chlorella sorokiniana, Scenedesmus subspicatus, Rhizoctonia solani*, and *Pythium ultimum* at 40 μg per disk. - no cytotoxicity on against brine shrimp at a concentration 10 μg/mL (24 h)	Red Sea	[25]
*Sarcophyton ehrenbergi*	Methylene chloride:methanol/room temperature	Sarcoehrenbergilid A–C (**39**–**41**)	5-Membered lactone	Moderate anti-proliferative activities against two human tumor cell lines: lung (A549) (IC_50_ = 50.1 − 76.4 μM), and liver (HepG2) (IC_50_ = 53.8 μM, only for sarcoehrenbergilid C (**41**)), and weak activity against colon (Caco-2) (IC_50_ > 100 μM).	Hurghada (Egyptian Red Sea costal)	[20]
					**South China Sea**	
*Sarcophyton* sp.	Methanol/room temperature	16-Hydroxycembra-1,3,7,11-tetraene (**15**)	Isopropyl cembrane	Antibacterial activity against *Staphylococcus aureus* (MBC and MIC values were 75 μg/mL and 25 μg/ mL, respectively. The MBC/MIC ratio was calculated to be 3.0 which indicated that the compound exhibits bactericidal activity	Karah Island, Terengganu, West Malaysia (5°35′52.6′’N,103°03′47.0E)	[21]
*Sarcophyton elegans*	Ethanol/room temperature	Sarelengans A and B (**44** and **45**) The cembranoids sarelengans C–G (**46**–**50**)	- Biscembranoids - (**46**): furanocembranoid; (**47**) and (**50**): 7-membered lactone; (**48**) and (**49**): 6-membered lactone	Sarelengan B (**45**) and sarelengan C (**46**) showed moderate inhibitory activities on nitric oxide production in RAW264.7 macrophages, with IC_50_ values being at 18.2 and 32.5 μM, respectively	Coast of Xisha Island	[22]
*Sarcophyton subviride*	Acetone/room temperature	The biscembranoid-like compounds bissubvilides A (**73**) and B (**74**)	Biscembranoids	These two molecules did not exert any cytotoxicity against human osteosarcoma MG-63 (IC_50_ > 30 μM) or A549 lung cancer (IC_50_ > 25 μM) cells or Huh7 human hepatology cancer stem cells (IC_50_ > 50 μM)	Coast of Xisha Island	[26]
*Sarcophyton ehrenbergi*	Acetone/room temperature	Sarcophytonoxides A–E (**31**–**35**)	Furanocembranoids	All of the cembranoids were inactive against the human ovarian cancer cell line A2780 (IC_50_ > 25 μM)	North Reef (Beijiao), Xisha Islands	[27]
*Sarcophyton stellatum*	Ethyl acetate/not reported	Stellatumolides A–C (**52**–**54**) Stellatumonins A (**55**) and B (**56**) Stellatumonone (**57**)	- 5-Membered lactone - Furanocembranoids - Isopropyl/isopropenyl acid cembranes	Only (+)-sarcophine (**61**) able to reduce the expression of cyclooxygenase-2 (COX-2) at 25–100 μM, and iNOS in LPS-stimulated RAW264.7 cells, at 50 and 100 μM, better nonselective COX-2 inhibitor than ibuprofen and aspirin, but less effective than the selective COX-2 inhibitor celecoxib. Absence of anti-cancer activity (HepG2, MDA-MB231 and A549 cell lines) of all compounds (IC_50_ > 20 μg/mL)	Dongsha Atoll, Taiwan	[33]
*Sarcophyton trocheliophorum*	Acetone/room temperature	Bicyclic cembranoids sarcophytrols M–U (**82**–**90**)	Isopropyl cembranes	No inhibitory activity against human protein tyrosine phosphatase 1B (PTP1B) enzyme, target for the treatment of type 2 diabetes and obesity. No cytotoxicities against the human tumor cell lines HL-60 and K-562, nor antibacterial activity against *Pseudomonas aeruginosa.*	Yalong Bay, Hainan Province	[23]
*Sarcophyton trocheliophorum*	Acetone/room temperature	Sarcophytonolides S-U (**91**–**93**) Sartrolides H-J (**94**–**96**)	- Isopropyl cembranes - Isopropyl cembranes	Sartrolide H (94) and 4*Z*,12*Z*,14*E*-sarcophytolide (**98**) had moderate inhibitory activity against PTP1B enzyme with IC_50_ = 19.9 and 15.4 μM, respectively, significantly less than the positive control, oleanolic acid (IC_50_ = 2.6 μM). 4*Z*,12*Z*,14*E*-Sarcophytolide (**98**) had moderate inhibitory activity against *Staphylococcus aureus* Newman strain (MIC_50_ = 250 μM)	Yalong Bay, Hainan Province	[34]
					**Philippine Sea**	
*Sarcophyton cherbonnieri*	Ethyl acetate/not reported	Cherbonolides A-E (**104**–**108**) Bischerbolide peroxide (**109**)	- 5-Membered lactone - Biscembranoids	Bischerbolide peroxide (**109**) exhibited the highest capacity for inhibiting the generation of superoxide anions (IC_50_ = 26.2 μM) Bischerbolide peroxide (**109**), cherbonolide A (**104**) and cherbonolide C (**106**) exhibited moderate activity on elastase release at 30 μM	Jihui Fish Port, Taiwan	[35]
					**Indian Ocean**	
*Sarcophyton stellatum*	Methanol/room temperature	(+)-Enantiomer of the cembranoid (1*E*,3*E*,11*E*)-7,8-epoxycembra-1,3,11,15-tetraene (**68**)	Isopropyl cembrane	Not determined, only the crude methanol extract. This showed moderate antimalarial activity (FCM29 strain of *Plasmodium falciparum*): IC_50_ = 35.20 μg/mL	Inner reef of Mohambo, Tamatave province, the east coast of Madagascar (17°29′15.0′’S, 49°28′32.1′’E)	[24]
					**Celebes Sea**	
*Sarcophyton* sp.	Ethanol/not reported	2-Hydroxy-crassocolide E (**19**)	5-Membered lactone	It exhibited cytotoxic activity against human breast tumor cell lines MCF-7 (IG_50_ = 18.13 ppm)	Mahengetang Island (Indonesia)	[31]
*Sarcophyton* sp.	Methanol/not reported	1*S*,2*E*,4*R*,6*E*,8*S*,11*S*,12*S*)-11,12-epoxy-8-hydroperoxy-4-hydroxy-2,6-cembradiene (**30**)	Isopropyl cembrane	It did not exhibit cytotoxic activity against human promyelocytic leukemia cells (HL-60) (IC_50_ > 30 μg/mL) It was not able to prevent the accumulation of NO, PGE_2_ and pro-inflammatory cytokines (TNF-α, IL-1β and IL-6) in LPS-induced RAW 264.7 cells, that is, it did not possess anti-inflammatory activity It had strong activity against the seaweed pathogens *Alteromonas* sp., *Cytophaga-Flavobacterium* and *Vibrio* sp.	Bohey Dulang, Sabah, Malaysia	[32]

**Table 3:** Harvesting locations of the soft corals of the genus *Sinularia*, extraction solvent, new compounds identified and their biological properties.

Soft Coral	Extraction	New Bioactive Cembranoid Diterpene	Type	Biological Activities	Location	Reference
					**South China Sea**	
*Sinularia* sp.	Methanol/room temperature	Sinularolide F (**128**)	5-Membered lactone	It showed potential anti-inflammatory activities against LPS-stimulated RAW 264.7 with IC_50_ values less than 6.25 μg/mL It exhibited anticancer activity against HL60 cell lines	Mantanani Island, Sabah	[42]
*Sinularia* sp.	Methanol using ultrasound/room temperature	Sinulins C and D (**132**) and (**133**)	Furanocembranoids	Sinulin D (**133**) showed mild target inhibitory activities against PTP1B (IC_50_ = 47.5 mM) positive control (sodium orthovanadate IC_50_ = 881 μM)	Yongxing Island	[43]
*Sinularia compacta*	Ethanol and then methylene chloride:methanol (1:1)/room temperature	Lobomichaolide (**117**), michaolide F (**118**), 20-acetylsinularolide B (**119**)	- 5-Membered lactone - 5-Membered lactone - 5-Membered lactone	Michaolide F (**118**) and 20-acetylsinularolide B (**119**) exhibited lethality toward brine shrimp *Artemia salina* with lethal ratios of 90.5% and 90.0% at a concentration of 50 μg/mL, respectively	Tongguling National Nature Reserve of Coral Reefs	[38]
*Sinularia erecta*	Ethyl acetate/not reported	Norcembranoids sinulerectols A (**110**) and B (**111**), a cembranoid sinulerectol C (**112**), and a degraded cembranoid sinulerectadione (**113**)	- (**110**): Isopropenyl cembrane - (**111**): Isopropenyl acid cembrane - (**112**): Isopropenyl cembrane	- Sinulerectadione (**113**) exhibited cytotoxicity toward K-562 and MOLT-4 cancer cell lines with IC_50_ values of 8.6 and 9.7 ± 2.9 μM, respectively. Sinulerectol C (**112**) showed cytotoxicity toward the K-562 cell line with an IC_50_ value of 9.2 μM. - Sinulerectols A (**110**) and B (**111**) exhibited potent anti-inflammatory activities in the inhibition of superoxide generation and elastase release. - Sinulerectol C (**112**) only exhibited significant activity in inhibiting elastase release	Coast of Dongsha Atoll	[37]
*Sinularia flexibilis*	Methanol/not reported	Epoxycembrane A (**155**)	Isopropenyl cembrane	Antifouling activity against the bryozoan *Bugula neritina* and the barnacle *Balanus albicostatus* (EC_50_ = 21.37 and 30.60 μg/mL, respectively)	Sanya Bay, Hainan Island	[39]
*Sinularia flexibilis*	Ethyl acetate/not reported	- Flexibilisins D and E (**146**) and (**147**) - Secoflexibilisolides A and B (**148**) and (**149**) - Flexibilisolide H (**150**)	- Isopropenyl cembrane - Seco cembrane derivatives - 7-Membered lactone	Non-toxic towards selective P-388, and HT-29 cancer cell lines No antioxidant activity No anti-inflammatory activity	Coast of Liuqiu, Taiwan	[45]
*Sinularia flexibilis*	Methanol/not reported	- *ent*-Sinuflexibilin D (**141**)	6-Membered lactone	It was active against adult T-cell leukemia (ATL), S1T cells It was active against against three strains of marine fungi *Exophiala* sp. NJM 1551, *Lagenidium thermophilum* IPMB 1401 and *Haliphthoros sabahensis* IPMB 1402 (MIC = 25, 25 and 50 μg/mL, respectively). MIC positive control (itraconazole) = 3.2 μg/mL	Mengalum Island, Sabah	[44]
*Sinularia flexibilis*	Methanol/not reported	- Sinulaflexiolides L-O (**151**–**154**) - *ent*-Sinuflexibilin D (**141**)	-6-Membered lactone -6-Membered lactone	Inhibitory activity of new cembranoids on LPS-induced NO production and the levels of TNF-á in RAW 264.7 macrophages under non-toxic concentrations (25 ìM): 16–33% and 26–53%, respectively	Yalong bay, Sanya in Hainan province, China	[46]

**Table 4:** Harvesting locations of the soft corals of the genus *Lobophytum*, extraction solvent, new compounds identified and their biological properties.

Soft Coral.	Extraction	New Bioactive Cembranoid Diterpene	Type	Biological Activities	Location	Reference
					**Indian Ocean**	
*Lobophytum crassum*	Methanol/room temperature	No cembranoids were isolated		Moderate activity of the crude methanol extract against the malarial parasite FCM29 strain of *Plasmodium falciparum* (IC_50_ value of 33.15 μg/mL)	Inner reef of Mohambo, Tamatave province, the east coast of Madagascar (17º29′15.0′’S, 49º28′32.1′’E)	[24]
					**Red Sea Coast**	
*Lobophytum* sp.	Chloroform:methanol (1:1)/room temperature	Cembrene A (**190**) (this is not new, but was the sole that presented biological activity among several metabolites)	Isopropenyl cembrane	Moderate antibacterial activity against *Acinetobacter* sp., *E.coli*, *Klebsiella pneumonia*, *Pseudomonas aeruginosa*, *Staphylococcus aureus*, *Staphylococcus epidermidis*, *Streptococcus pneumonia* - High toxicity against brine shrimp *Artemia salina* (LD_50_ = 25 μg/mL) - Antitumor activity against Erhlich carcinoma cells (LD_50_ = 50 μg/mL, respectively	Saudi Arabia Red Sea Coast at Jeddah	[52]
					**South China Sea**	
*Lobophytum crassum*	Ethyl acetate/not reported	Lobophylins F-H (**180**–**182**)	Isopropenyl cembrane	Not evaluated	Coast of Dongsha Atoll	[56]
*Lobophytum crassum*	Ethyl acetate/not reported	Lobophyolide A and B (**174**) and (**175**)	5-Membered lactone	- Both (<50 μg/mL) presented a potent inhibitory effect on IL-12 and NO release (inhibition rates of >90%) in LPS-activated dendritic cells - Lobophyolide A (**174**) and B (**175**) also had considerable cytotoxicity with survival percentage of dendritic cells, under the concentration of 50 μg/mL, of 76 and 52, respectively.	Coast of Pingtung, Taiwan	[9]
*Lobophytum crassum*	Methanol/not reported	Locrassumins A,B, D-G (159),(**160**), (**161**)-(**164**), locrassumin C (**168**) (–)-laevigatol B (**165**), (–)-isosarcophine (**166**) (–)-7*R*,8*S*-dihydroxydeepoxysarcophytoxide (**167**)	Locrassumins A-C, E: Isopropyl cembrane; Locrassumin D: 7-membered lactone; Locrassumin F and (–)-isosarcophine: 5-membered lactone; Locrassumin G, (–)-laevigatol B and (–)-7*R*,8*S*-dihydroxydeepoxysarcophytoxide: Furanocembranoid	Locrassumins A (**159**) and G (**164**), (**169**), (**170**) and (**172**) showed moderate inhibition against LPS-induced NO production in mouse peritoneal macrophages with IC_50_ values of 17 and 13 μM, 24, 8 and 17 μM, respectively. No inhibitory effect was observed for the other compounds (IC_50_ > 30 μM)	Inner coral reef of Meishan, Hainan Province	[45]
					**East China Sea**	
*Lobophytum* sp.	Acetone/not reported	Compound **A** (a new rare casbane-tipe diterpenoid), two new cembrane diterpenoids (Compounds **B** and **C**)	- Casbane - Isopropyl cembranes	- Weak anti-bacterial activity (*Staphylococcus aureus*, *Salmonella enterica* and *E. coli*) - Moderate cytotoxicity against human colon cancer cells (HCT116) with IC_50_ values ranging from 135.57 to 177.11 μM - Anti-inflammatory activity in LPS/IFN-γ (LPS/interferon-γ)-stimulated RAW 264.7 macrophages cells (IC_50_ 41.21–74.76 μM)	Irabu Island, Okinawa, Japan	[53]
					**Aquaculture**	
*Lobophytum crassum*	Ethyl acetate/not reported	Culobophylins D (**185**) and E (**186**)	Culobophylins D: Isopropenyl cembrane; Culobophylins E: Isopropenyl acid cembranes	13-Acetoxysarcocrassocolide (**187**), lobocrassin B (**186**) and 14-deoxycrassin (**142**) were active against diverse leukemia cell lines (K562, U937, Molt4, and Sup-T1) with IC_50_ values ranging from 1.2 to 7.1 μM	National Museum of Marine Biology and Aquarium (Pingtung, Taiwan)	[57]
